# Nitrostilbenes: Synthesis and Biological Evaluation as Potential Anti-Influenza Virus Agents

**DOI:** 10.3390/ph15091061

**Published:** 2022-08-26

**Authors:** Marta De Angelis, Barbara De Filippis, Marwa Balaha, Letizia Giampietro, Mariya Timotey Miteva, Giovanna De Chiara, Anna Teresa Palamara, Lucia Nencioni, Adriano Mollica

**Affiliations:** 1 Laboratory Affiliated to Institute, Department of Public Health and Infectious Diseases, Pasteur Italia-Cenci Bolognetti Foundation, Sapienza University of Rome, 00185 Rome, Italy; 2 Department of Pharmacy, University “G. d’Annunzio” University of Chieti-Pescara, via dei Vestini 31, 66100 Chieti, Italy; 3 Department of Pharmaceutical Chemistry, Faculty of Pharmacy, Kafrelsheikh University, Kafr El Sheikh 33516, Egypt; 4 Institute of Translational Pharmacology, National Research Council, 00133 Rome, Italy; 5 Department of Infectious Diseases, Italian National Institute of Health, 00161 Rome, Italy

**Keywords:** resveratrol, polyphenols, influenza virus, redox state, antivirals

## Abstract

Resveratrol (RSV) is a natural stilbene polyphenolic compound found in several plant species. It is characterized by antioxidant properties, and its role in controlling viral replication has been demonstrated for different viral infections. Despite its promising antiviral properties, RSV biological activity is limited by its low bioavailability and high metabolic rate. In this study, we optimized its structure by synthesizing new RSV derivatives that maintained the phenolic scaffold and contained different substitution patterns and evaluated their potential anti-influenza virus activity. The results showed that viral protein synthesis decreased 24 h post infection; particularly, the nitro-containing compounds strongly reduced viral replication. The molecules did not exert their antioxidant properties during infection; in fact, they were not able to rescue the virus-induced drop in GSH content or improve the antioxidant response mediated by the Nrf2 transcription factor and G6PD enzyme. Similar to what has already been reported for RSV, they interfered with the nuclear-cytoplasmic traffic of viral nucleoprotein, probably inhibiting cellular kinases involved in the regulation of specific steps of the virus life cycle. Overall, the data indicate that more lipophilic RSV derivatives have improved antiviral efficacy compared with RSV and open the way for new cell-targeted antiviral strategies.

## 1. Introduction

**Figure 1:**
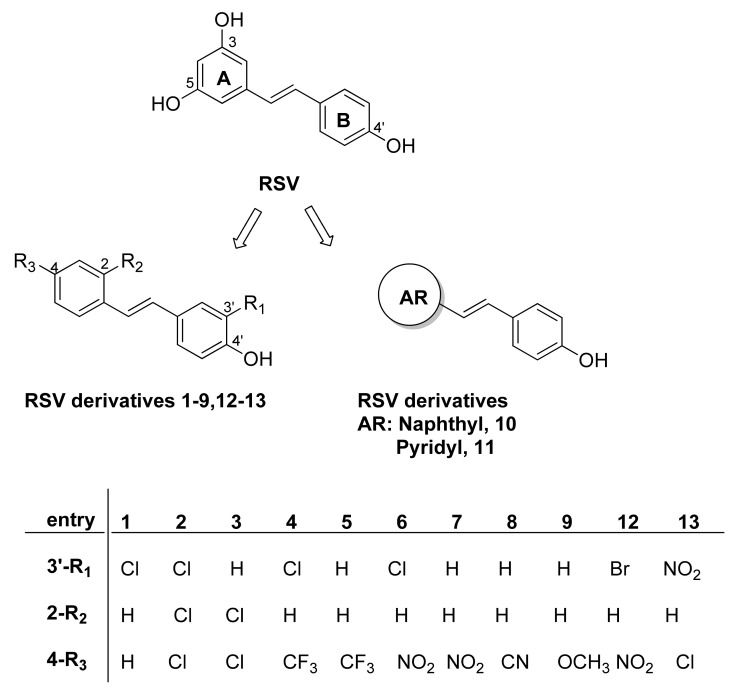
Resveratrol (RSV) and its derivatives **1**–**13**.

Resveratrol (RSV, 3,4′,5-trihydroxystilbene, Figure 1) is a well-known natural polyphenol belonging to the subclass of stilbenoids, which are synthesized by various plants, including peanuts, grapes, and blueberries, in response to physiological or stress stimuli and are present in red wine [1,2]. Many research studies conducted on RSV have demonstrated biological activity in in vitro and in vivo models and have highlighted the potential benefits of this agent for many diseases [3], such as cancer [4,5], cardiovascular disease [6], longevity [7,8], bone health [9,10], neurodegenerative [11], and infectious diseases, including those caused by DNA and RNA viruses [12,13,14,15,16]. RSV efficacy has been related to its ability to modulate several biochemical mediators, as described in many comprehensive review articles on the subject [17,18].

Similar to other phenolic compounds, the antiviral activity of RSV has been proven in several in vitro infection models. For example, RSV activity was reported against Epstein¬–Barr virus in Raji and human B cells [19], herpes simplex-1 virus in VERO and MRC-5 cells [20], respiratory syncytial virus in lung epithelial cells [21], human immunodeficiency virus-1 in primary peripheral blood lymphocytes [22], and dengue virus infection [23]. In the case of the influenza A virus, our group demonstrated that RSV interfered with nuclear–cytoplasmic translocation of viral ribonucleoproteins through the inhibition of cellular kinases, but it was not associated with an increase in the GSH-mediated antioxidant response [12]. Moreover, we observed the anti-influenza virus properties of analogues using an in vitro model [24]. More recently, the potential antiviral activity of RSV has been suggested against SARS-CoV-2 infection [25,26,27]. We also tested a mixture of polyphenols and RSV precursors in influenza virus and SARS-CoV-2 infection models, demonstrating their efficacy in inhibiting viral replication in both models of respiratory virus infections [28].

Despite the potential antiviral activity of RSV, it is characterized by poor bioavailability and a high metabolic rate in the gut and liver, which lead to a low plasma concentration [29]. To overcome this drawback, many efforts have been directed at the synthesis of compounds structurally related to RSV [30] and/or the development of nanoformulations [31] that possess different pharmacological profiles, including antiviral activities [32].

In continuation of our work, we investigated the potential anti-influenza virus activity of a series of RSV derivatives containing different substitution patterns. The synthesis of two novel nitrostilbene compounds was also reported.

## 2. Results

### 2.1. Chemistry

In our ongoing study on the structural optimization of RSV, we focused on the R_2_ and R_4_ groups in the new scaffold and maintained the 4′-OH group on ring B (Figure 1) due to its well-documented role in antioxidant activity [33,34]. We started from the evidence that the introduction of a substituent with different electronic and lipophilic properties at the 4-position on the aromatic ring A (R_3_) (Figure 1) improved the anticancer, antibacterial, and antioxidant activities [35,36,37,38].

In order to expand our knowledge on the antiviral potential of our RSV derivatives, we selected some of these compounds (**1**–**11**, Figure 1) to evaluate their activity against the influenza virus. In the second step, we synthesized two new compounds with a nitro group on aromatic ring A (**12**) or B (**13**) to include in the study. The tested compounds contained the stilbene scaffold, and the 4′-phenol (ring B) was maintained because of the importance of this group to antiviral activity, as highlighted by our previous work [24]. Structural variations were present on aromatic ring A, where the resorcinol group of RSV was replaced by the aromatic ring with a substituent at the 2- and/or 4-position (compounds **1**–**9** and **12**–**13**) or was replaced by a naphthyl or a pyridyl moiety (compounds **10**–**11**). In some cases, a chlorine (**1**, **2**, **4**, and **6**), bromine (**12**), or nitro group (**13**) was inserted at the 3′-position. The structures of the tested compounds are shown in Figure 1. 

**Figure 2:**
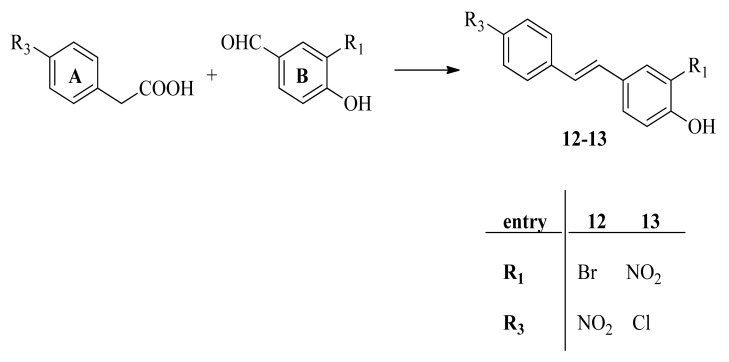
Synthesis of compounds **12**–**13**. Reagents and conditions: 4-hydroxybenzaldehyde (1.0 eq.), aryl acetic acid (1.2 eq.), piperidine (2.5 eq.); 130 °C, 20–23 h.

The synthesis of compounds **1**–**11** was carried out following the reported route [35,38]. The new compounds, compounds **12**–**13,** were obtained as shown in Figure 2. The corresponding 4-hydroxybenzaldehyde and the appropriate aryl acetic acid were mixed in the presence of piperidine at 130 °C. The usual aqueous work-up and purification using silica gel column chromatography produced the desired phenols. Confirmation of the structures and purity of compounds **12**–**13** was obtained from their ^1^H and ^13^C-NMR spectra and by a comparison of the signals with those reported in the literature [39].

### 2.2. Antiviral Activity of RSV Derivatives against Influenza A Virus Infection

RSV derivatives **1**–**11** were tested in vitro for their antiviral activity against influenza A Puerto Rico 8/34/H1N1 (PR8) virus. First, we evaluated the cytotoxicity of the compounds on a lung epithelial cell line (A549) permissive to PR8 virus infection. On the basis of our previous studies [12,28], we used concentrations of the RSV derivatives ranging from 10 to 40 μg/mL. As shown in Table 1, compounds **2**, **3**, and **4** caused a 50% reduction in cell viability (CC50) at concentrations of 39.5, 30.3, and 38.8 μg/mL, respectively, while other compounds were toxic only at higher concentrations. Afterward, cells were infected with the influenza virus as described in the methods section, treated with the above concentrations of compounds **1**–**11** for 24 h, and then stained with anti-hemagglutinin (anti-HA) antibody. The antiviral activity of RSV derivatives was evaluated by an In-Cell Western (ICW) assay. All the RSV derivatives caused a reduction in HA expression and low IC50 (50% inhibitory concentration) values (except for compounds **10** and **11**). Furthermore, compounds **6** and **7** showed higher selectivity indexes (SIs) of 12.4 and 5.9 μg/mL, respectively, and they were selected for the next studies.

### 2.3. RSV Derivatives ***6***, ***7***, ***12***, and ***13*** Strongly Reduce Viral Protein Synthesis and Viral Particle Production

**Figure 3:**
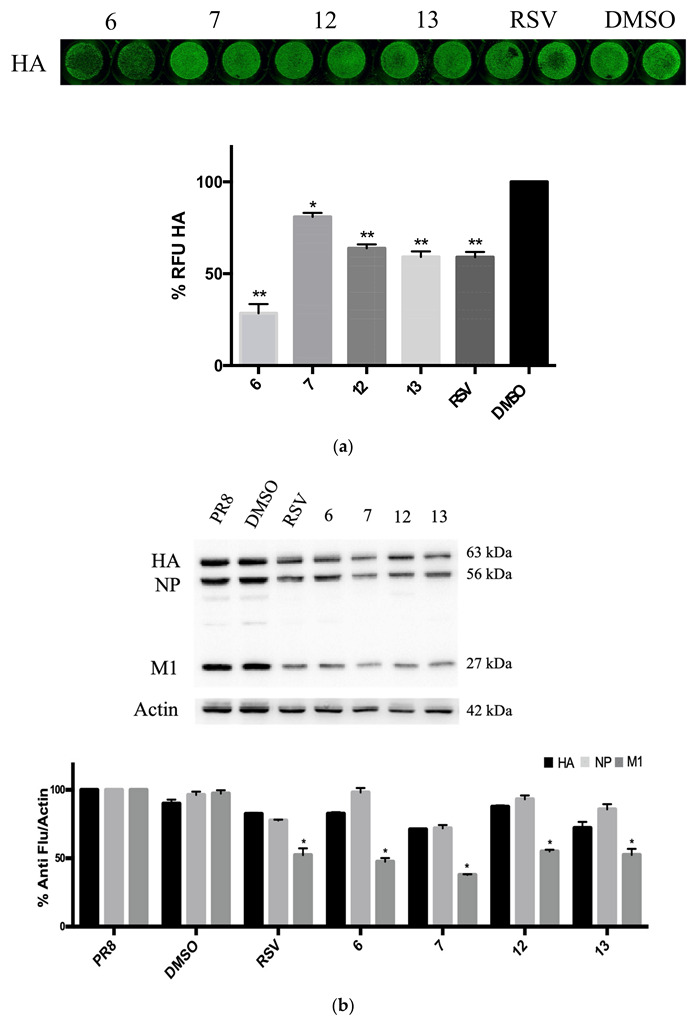
RSV derivatives **6**, **7**, **12**, and **13** decrease viral proteins synthesis. (**a**) In-Cell Western assay of hemagglutinin (HA) protein expression in influenza A PR/8/H1N1 virus-infected A549 cells treated with the RSV derivatives (compounds **6**, **7**, **12**, and **13**) or with RSV at a concentration of 20 μg/mL. Cells treated with solvent (DMSO) were used as control. The graph represents the percentage (%) of the Relative Fluorescence Unit (RFU) obtained from viral HA protein expression compared with DMSO (considered 100%). Data are expressed as means ± S.D. of two experiments, each performed in duplicate (*n* = 4) (* *p* < 0.05, ** *p* < 0.001 vs. DMSO-treated condition). (**b**) Western blot analysis of influenza A virus proteins, hemagglutinin (HA), nucleoprotein (NP), or matrix protein 1 (M1) immunostained with anti-Flu. Actin was used as loading control. The blot shown is one representative of three performed. In the panel below, densitometry analysis of HA, NP, and M1 protein expression is shown. Data are expressed as ratio of each viral protein to actin (* *p* < 0.05 vs. PR8).

On the basis of the IC50 values, we decided to deeply investigate the mechanism of action exerted by compounds **6** and **7**. These compounds contain a nitro group at the R4 position. Recently, Saúl Noriega et al. conducted a large analysis on the role of nitro function in medicinal chemistry [40]. The nitro group has a high electron-withdrawing ability by means of both resonance and inductive properties, [41] affecting the biological and pharmacokinetic profiles. Nitro-containing compounds represent a useful and privileged moiety in medicinal chemistry, showing a wide range of biological activities, such as antihypertensive, antineoplastic, antiparasitic, antibiotic, and tranquilizer activities [41]. Nitro-drugs have been studied for their antiviral properties, and some of them are employed as medication [42,43,44]. For this reason, two new analogues, compounds **6** and **7**, were synthesized and added to the study to evaluate the role of the position of the nitro group (3′-NO_2_ in **13** vs. 4-NO_2_ in **6** and **7**) and the type of halogen (3′-Cl in **6** vs. 3′-Br in **12**) on their activity. The new compounds, compounds **12** and **13,** did not show cytotoxicity on the A549 cell monolayer (data not shown), and we decided to select the concentration of 20 μg/mL for the next antiviral assays according to our previous results obtained with the unmodified RSV [12]. First, we evaluated the HA protein expression by ICW assay on cell monolayer infected with PR8 virus and treated with RSV derivatives for 24 h post-infection (p.i.). As shown in Figure 3a, the HA expression was reduced with all the tested compounds. In particular, compound **6** showed higher efficacy in reducing HA expression (75% of inhibition) on infected cell monolayers compared with RSV and the other derivatives. Western blot analysis of influenza virus protein synthesis confirmed the inhibition of HA expression but also showed a decrease in the early viral nucleoprotein (NP) expression with compounds **6**, **7**, and **13**. Interestingly, a strong reduction in the late viral protein matrix 1 (M1) was observed in cells treated with all the tested compounds (Figure 3b).

**Figure 4:**
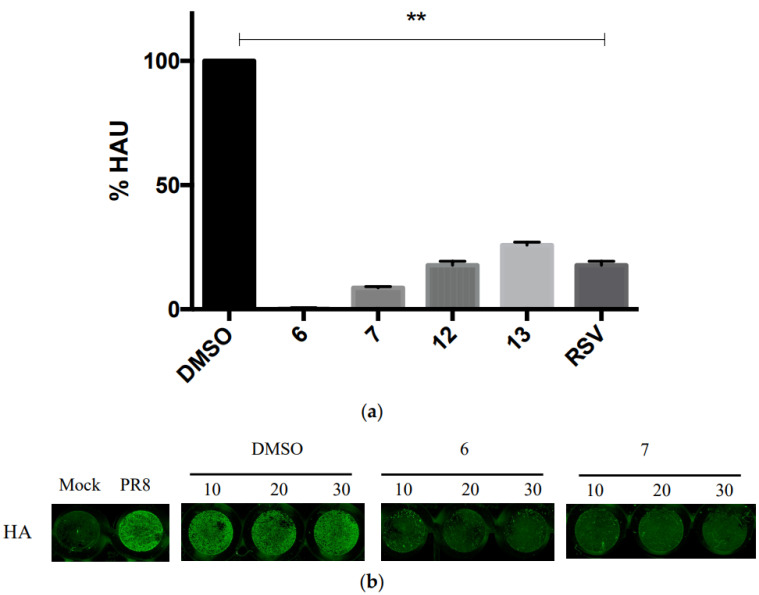
RSV derivatives **6** and **7** impair viral production and reduce viral infectivity. (**a**) Hemagglutination assay (HAU) on supernatants of A549 cells infected with influenza A PR/8/H1N1 virus and treated with the RSV derivatives (compounds **6**, **7**, **12**, and **13**), or with RSV at a concentration of 20 μg/mL. Cells treated with the solvent (DMSO) were used as control. Data are expressed as percentage (%) of HAU and represent the mean ± S.D. of three separate experiments, each performed in duplicate (*n* = 6) (** *p* < 0.001 vs. DMSO treated condition). (**b**) ICW assay of hemagglutinin (HA) protein expression on MDCK cells infected with supernatants obtained from A549 cells treated with different concentrations (range: 10–30 μg/mL) of RSV derivatives. Images represent one experiment of two performed.

Next, we quantified the viral particles released in the supernatants of A549 cells treated with compounds **6** and **7** and analogues **12** and **13** using the hemagglutination assay (HAU). Although all RSV derivatives were effective, the results showed that compounds **6** and **7** markedly decreased the viral titer, also in comparison with RSV, with a pronounced effect mediated by compound 6 (Figure 4a). Thus, we evaluated whether the viral particles released from A549 infected and treated cells with compounds **6** and **7** (concentration range: 10 to 30 μg/mL of RSV derivatives) were still infective. To this aim, the harvested supernatants were used to newly infect fresh MDCK cell monolayers. After 24 h, cells were stained with anti-HA antibodies and analyzed by ICW. As shown in Figure 4b, the HA protein expression on infected cells was markedly reduced with both compounds **6** and **7**, indicating that the supernatants of treated cells contained a lower number of viral particles that were no longer able to efficiently infect new cells and confirming the potential antiviral properties of compounds **6** and **7**.

### 2.4. Compounds ***6***, ***7***, ***12***, and ***13*** Do Not Rescue the GSH-Mediated Antioxidant Response

**Figure 5:**
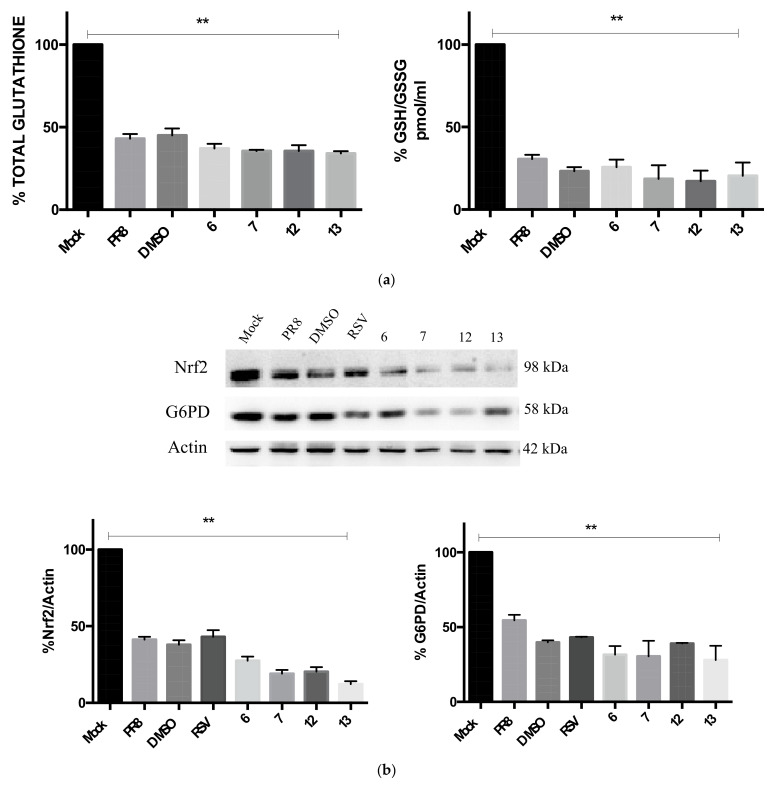
Compounds **6**, **7**, **12**, and **13** do not rescue the GSH-mediated antioxidant response. (**a**) Total glutathione (left graph) and GSH/GSSG ratio (right graph) measured in A549 cells after 24 h of influenza A/PR/8/H1N1 virus infection and treatment with RSV derivatives. The analysis was performed by using a colorimetric assay as described in the methods, and the results are represented as percentages (%) with respect to mock cells. Data are expressed as the means ± S.D. of three experiments, each performed in duplicate (*n* = 6) (** *p* < 0.001 vs. mock). (**b**) Western blot analysis of Nrf2 and G6PD protein expression in A549 cells infected with influenza A/PR/8/H1N1 virus and treated for 24 h. Actin was used as loading control. The panel below represents the densitometry analysis of both proteins normalized to actin. Data are expressed as means ± S.D. of three experiments performed (** *p* < 0.001 vs. mock).

It is known that influenza virus infection induces redox state changes in host cells by increasing reactive oxygen species (ROS) production and reducing the GSH content as well as the Nrf2-mediated antioxidant response [45,46]. These alterations can, in turn, activate some redox-sensitive pathways useful for influenza virus replication [47]. In the present study, we wanted to explore whether the RSV derivatives inhibited viral protein synthesis and viral particle production by restoring the intracellular reducing conditions. To this aim, we measured the intracellular content of total and reduced GSH after treatment with compounds **6**, **7**, **12**, and **13**. As shown in Figure 5a, the GSH was significantly decreased after 24 h of infection, in line with the data obtained in our previous work [12,46,47], but treatment with all RSV derivatives was not able to rescue the level of GSH. These data suggest that the inhibitory activity of these compounds is not related to the modulation of the GSH-mediated antioxidant response. Additionally, we found that the virus-induced downmodulation of Nrf2 and G6PD protein expression was not upregulated after the treatment (Figure 5b).

### 2.5. Compounds ***6*** and ***7*** Impair Viral Replication by Blocking the Viral NP Protein in the Nuclear Compartment

**Figure 6:**
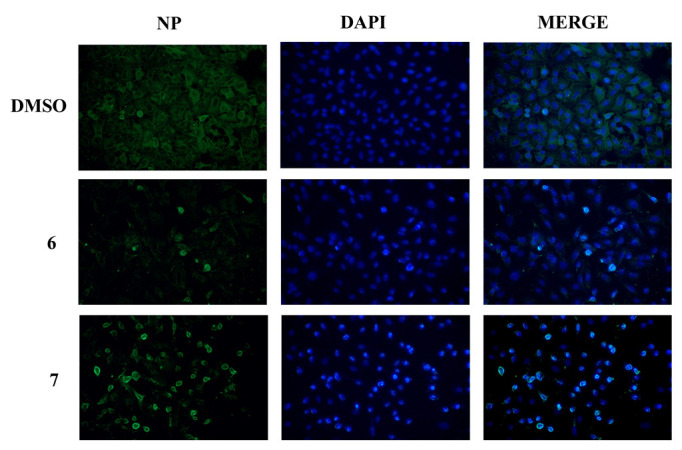
Compounds **6** and **7** retain viral NP in the nucleus. Immunofluorescence analysis of viral nucleoprotein (NP, green) in A549 cells infected for 24 h with influenza A/PR/8/H1N1 virus and treated with compounds **6**, **7**, or DMSO solvent. Nuclei were stained with DAPI (blue). Images represent one experiment of two performed.

Considering the obtained results, we identified compounds **6** and **7** as the more promising RSV derivatives because they were more effective in reducing viral particle production (Figure 4). Since RSV was shown to inhibit the nuclear–cytoplasmic traffic of viral NP by impairing specific cellular kinases [12], we evaluated the NP nuclear–cytoplasm translocation in infected cells treated or not treated with compounds **6** and **7** by immunofluorescence analysis**.** As shown in Figure 6, after 24 h of infection, the NP was located predominantly in the cytoplasm in infected cells treated only with the solvent DMSO. On the contrary, in treated cells, NP was still localized in the nuclei, and the NP export was inhibited. Furthermore, a smaller number of NP-positive cells was observed in cells treated with compound **6**, suggesting a low quantity of viral particles spreading from cells and the higher efficacy of this RSV derivative in inhibiting the influenza virus replicative cycle (Figure 6, middle panel).

Generally, the results indicate the antiviral properties of RSV derivatives and suggest an impairment of specific steps of the virus life cycle, likely by inhibiting cell pathways involved in the regulation of viral replication [12,46,47,48,49] rather than modulating the intracellular redox state.

## 3. Discussion

In the present work, we demonstrated the efficient antiviral activity of newly synthesized RSV derivatives. The study started from our previous data, which demonstrated that RSV inhibited influenza virus replication in vitro and in vivo [12], and we demonstrated that RSV precursors, such as polydatin, can also exert anti-influenza virus properties [28]. However, the poor bioavailability and the high metabolic rate of RSV require the identification of new compounds with optimized structures. In previous studies, we have shown that the substitution of the 4-position on the aromatic ring of RSV with groups with different electronic and lipophilic properties improved their biological activity [35,36,37,38]. Most of the compounds studied here were tested for their antioxidant activity, and a correlation between the presence of 3′-chlorine and an improved scavenger ability of 4′-hydroxyl [38] was proven. Research conducted using HPLC showed that most of the compounds tested in this study had values of Log P higher than those of RSV [38]. This could be associated with the presence of two or three chlorine atoms or a trifluoromethyl group. Starting from these results, we hypothesized that all tested compounds (**1**–**13)** would have higher lipophilia and a potential ability to affect viral structures or to enter the cell more easily and inhibit specific steps of the virus life cycle.

Moreover, in one of our previous studies, we proved the importance of phenolic groups in interfering with two intracellular key steps of the influenza virus life cycle, such as the MAP kinases that control the nuclear–cytoplasmic traffic of viral ribonucleoprotein complex and the redox-sensitive pathways essential for the maturation of viral hemagglutinin protein [24]. The combination of these two structural characteristics could promote the activity of all the RSV derivatives, inhibiting the expression of HA viral protein with lower IC50 values compared with the reference compound (Table 1).

Then, we wanted to explore the possible antiviral effects of compounds **1**–**11** and two new nitro-derivatives, compounds **12**–**13,** against influenza virus infection.

Furthermore, due to the lower IC50 value and the higher SI (12.4 and 5.9, respectively) of compounds **6** and **7**, we decided to focus our attention on these two derivatives. They are characterized by a 4-nitrophenyl substituent instead of the hydroxyl group of RSV and, in the case of compound **6,** by a chlorine in 3′-position (Figure 1). In the literature, it is reported that the increase in lipophilicity due to the introduction of a halogen atom to one or more specific positions of a biologically active molecule leads to a higher partitioning of the halogenated compound into the lipophilic portion of a cell membrane or lipophilic domains of a target [50,51]. Moreover, the electronic property of the nitro group could affect biological activity [38]. To explore the role and the position of the nitro group and the type of halogen, we aimed to study two new analogues in which the nitro group was shifted from the 4-position to the 3′-position (compound **13**) and the 3′-bromine instead of the 3′-chlorine atom (compound **12**). Both were added to the study and demonstrated efficacy in reducing the synthesis of viral proteins. However, these kinds of chemical modifications did not improve the antiviral activity of **6** and **7**. Indeed, although all the RSV derivatives reduced the expression of viral proteins compared with the DMSO treated condition, only compounds **6** and **7** drastically reduced the viral particle production with respect to unmodified RSV treatment, as reported in Figure 4a,b, with a pronounced antiviral effect reported by compound **6**.

The GSH depletion and the induction of oxidative stress condition is a main feature of influenza virus infection, and it has been well documented by different studies [12,47]. Moreover, the infection decreases the antioxidant response mediated by the Nrf2 pathway and the G6PD enzyme, which is involved in the regeneration of GSH [46]. Although RSV exerts antioxidant and free radical-scavenger properties [52,53], and it has been reported to increase GSH levels in different experimental models [54,55], in our previous work, RSV treatment did not rescue the GSH level in infected conditions [12]. Considering this evidence, we wanted to explore whether the new RSV derivatives acted through the same mechanism. The influenza virus infection reduced the GSH/GSSG ratio as expected, but the RSV derivative treatment did not increase the reducing condition in the host cells. Indeed, Nrf2 and G6PD expression, which was found to decrease during the infection, was not restored after the treatment either. These findings may be related to the free radical scavenger activity of RSV itself, which, on the one hand, can donate hydrogen atoms and thus eliminate reactive species but, on the other hand, can generate phenoxyl radicals that are able to oxidize the GSH [56]. Moreover, the configuration and substitution of the RSV structure, as well as the concentration and the time of exposure of the infected cells to this compound, can influence several antioxidant mechanisms and pathways [57,58]. The substitution of the resorcinol moiety of RSV with the 4-nitro or 4-chloro phenyl moiety (ring A) negatively affected the antioxidant activity. Moreover, neither the type of halogen (Cl vs. Br) nor the position of the nitro group had an influence on the activity, confirming that the mechanism of action cannot be attributed to their antioxidant properties.

We previously reported that the antiviral effect of RSV was related to the inhibition of c-Jun N-terminal kinase (JNK) and p38 MAPK pathways, which are involved in regulating viral ribonucleoprotein (vRNP) nuclear–cytoplasm traffic during influenza virus replication [47]. In line with this evidence, we observed that compounds **6** and **7** interfered with the nuclear export of NP, as shown by the immunofluorescence analysis of NP in treated cells (Figure 6). Altogether, these data demonstrate that compound **6** was the most effective antiviral RSV derivative and acted by causing a drastic reduction in influenza virus titer and exerting a remarkable ability to block the NP viral protein traffic.

Although the data identify promising antiviral agents for the treatment of influenza virus infection, further studies are necessary to better understand the biological effects of nitrostilbenes in blocking specific steps of the virus replicative cycle as well as to evaluate the modulation of cell kinases involved in the regulation of NP traffic during the treatment.

## 4. Materials and Methods

### 4.1. Chemistry

Melting points were determined with a Buchi Melting Point B-450. ^1^H and ^13^C-NMR spectra were recorded on a Varian Mercury 300 spectrometer. Proton chemical shifts referred to the TMS internal standard. Chemical shifts are reported in parts per million (ppm, δ units). Coupling constants are reported in units of Hertz (Hz). Splitting patterns are designated as s, singlet; d, doublet; and dd, double doublet. All commercial chemicals and solvents were reagent grade and were used without further purification unless otherwise specified. All reactions were carried out with the use of the standard techniques and were monitored by thin-layer chromatography on silica gel plates (60F-254, E. Merck, Merck Group, Darmstadt, Germany) and visualized with UV light. The spectra of compounds **12-13** are reported in Supplementary Material (Figures S1–S4).

#### 4.1.1. General Procedure for the Preparation of Phenols **12**–**13**

##### (E)-2-Bromo-4-(4-Nitrostyryl)Phenol, **12**

A stirred mixture of piperidine (2.5 eq., 210.74 mg), 4-hydroxy-2-bromobenzaldehyde (1.0 eq., 200 mg), and 4-nitrophenylacetic acid (1.2 eq., 215.20 mg) was heated gradually to 130 °C and allowed to react for 23 h. The residue was cooled to room temperature and partitioned between DCM (30 mL) and HCl 1N (4 × 15 mL). The organic phase was dried over Na_2_SO_4_ and concentrated under reduced pressure to yield the crude product, which was purified by column chromatography (eluent cyclohexane:ethyl acetate 1:1), yielding the pure phenol **12** as a yellow–brown solid (114.84 mg, 36.0%). M.p. 200.2–200.4 °C. ^1^H-NMR (C*D*Cl_3_) δ 6.99 (d, 1 H, C*H*CH, J = 16.2 Hz); 7.04 (d, 1 H, C*H*_Ar_, J = 9.0 Hz); 7.14 (d, 1 H, CHC*H*, J = 16.5 Hz); 7.42 (dd, 1 H, C*H*_Ar_, J_1-2_ = 2.4 Hz, J_2-3_ = 6.6 Hz); 7.59 (d, 2 H, C*H*_Ar_, J = 9.3 Hz); 7.68 (d, 1 H, C*H*_Ar_, J = 1.8 Hz); 8.21 (d, 2 H, C*H*_Ar_, J = 8.7 Hz); ^13^C-NMR (C*D*Cl_3_) δ 116.45, 123.68, 124.18, 125.50, 126.70, 127.80, 127.98, 129.57, 130.44, 130, 59, 131, 28, 131.78, 132.32, 143.64.

##### (E)-4-(4-Chlorostyryl)-2-Nitrophenol, **13**

A stirred mixture of piperidine (2.5 eq., 254.75 mg), 4-hydroxy-2-nitrobenzaldehyde (1.0 eq., 200 mg), and 4-chlorophenylacetic acid (1.2 eq., 407.36 mg) was heated gradually to 130 °C and allowed to react for 20 h. The residue was cooled at room temperature and partitioned between DCM (30 mL) and HCl 1N (4 × 15 mL). The organic phase was dried over Na_2_SO_4_ and concentrated under reduced pressure to yield the crude product, which was purified by column chromatography (eluent cyclohexane:ethyl acetate 1:1), yielding the pure phenol **13** as a yellow–brown solid (152.66 mg, 27.8%). M.p. 176.5–177.9 °C. ^1^H-NMR (C*D*Cl_3_) δ 6.99 (s, 2 H, C*H*C*H*); 7.16 (d, 1 H, C*H*_Ar_, J = 9.0 Hz); 7.33 (d, 1 H, C*H*_Ar_, J = 7.33 Hz); 7.42 (d, 2 H, C*H*_Ar_, J = 9.0 Hz); 7.7 (dd, 1 H, C*H*_Ar_, J_1-2_ = 2.4, J_2-3_= 6.6 Hz); 8.17 (d, 1 H, C*H*_Ar_, J = 2.4 Hz); ^13^C-NMR (C*D*Cl_3_) δ 120.38, 122.43, 126.21, 127.69, 128.42, 128.97, 130.03, 133.76, 135.02, 135.05, 154.50.

### 4.2. Biology

#### 4.2.1. Cell Cultures and Virus Production

Madin-Darby Canine Kidney epithelial cell line, MDCK (ATCC catalogue no. CCL-34) and human lung adenocarcinoma cell line, A549 (ATCC catalogue no. CCL-185) were grown in Minimum Essential Medium (MEM) and Dulbecco’s Modified Eagle Medium (DMEM), respectively, supplemented with 10% fetal bovine serum (FBS), 0.3 mg/mL glutamine, 100 U/mL penicillin, and 100 mg/mL streptomycin.

For virus production, influenza virus A/Puerto Rico/8/34 H1N1 (PR8 virus) strain was grown in the allantoic cavities of 11-day-old embryonated chicken eggs, harvested after 48 h of incubation at 37 °C, and centrifuged at 5000 rpm for 30 min to remove cellular debris. During the infection procedure, confluent monolayers of epithelial cells (A549 or MDCK) were challenged for 1 h at 37 °C with PR8 virus stock at a multiplicity of infection (m.o.i.) of 0.01 or with supernatants obtained from infected cells and incubated for 1 h at 37 °C. Then, cells were washed with PBS and incubated with a medium supplemented with 2% FBS. Mock infection was performed with the same dilution of allantoic fluid.

#### 4.2.2. Cytotoxicity Assay

The cytotoxicity of RSV derivatives was evaluated on A549 cells using MTT (3-(4,5-dimethylthiazol-2-yl)-2,5-diphenyl tetrazolium bromide) reagent (Sigma-Aldrich, St. Louis, MO, USA). In this assay, cells were seeded in a 96-well plate at a concentration of 2 × 10^4^/well in DMEM without phenol red and supplemented with 10% FBS. The compounds were dissolved in DMSO and added to the cell monolayer. After 24 h, 10 µL of the 5 mg/mL solution of MTT was added to the cell culture, which was incubated at 37 °C for 3 h. Then, the medium was solubilized in absolute isopropanol containing 0.1 N HCl. The absorbance of converted dye was measured in an ELISA plate reader at a wavelength of 590 nm. The results were used to calculate the 50% cytotoxic concentration (CC50), defined as the concentration of RSV derivatives required to reduce cell viability by 50% compared with the solvent-treated (DMSO) control condition.

#### 4.2.3. Cell Treatment

The selected concentration of RSV derivatives that were not toxic on the cell monolayer (10–40 μg/mL) were tested against PR8 virus infection. A549 cells were infected with the PR8 virus, and after 1 h of viral adsorption of the compounds, they were dissolved in DMSO and added to the cell culture for 24 h post-infection. The concentration of compounds causing a 50% reduction of viral infection (IC50) was used to identify the Selectivity Index (SI) of each RSV derivative, and it was calculated as the ratio CC50/IC50.

#### 4.2.4. Hemagglutination (HAU) Assay

Viral titration was performed by HAU assay. The test is based on the ability of the hemagglutinin (HA) protein of the influenza virus to bind the receptor (sialic acid) on cells, including erythrocytes, forming a clump of linked erythrocytes. When the amount of the virus is low, red blood cells (RBCs) are not linked to each other, and they precipitate at the bottom of the plate, forming a red button. In the test procedure, twofold serial dilutions of the supernatant obtained from infected cells were added to the wells of 96-well U-bottom plates and mixed with 0.5% of RBCs diluted in PBS and incubated at room temperature until the formation of the red button. The hemagglutinating unit (HAU) was identified as the virus dilution before red button formation.

#### 4.2.5. In-Cell Western Assay

A549 cells were grown in 96-well plates (2 × 10^4^ cells/well) and infected or mock-infected with the PR8 virus. Then, cells were washed in PBS and treated with RSV derivatives. After 24 h of infection and treatment, cells were fixed with 4% paraformaldehyde (Santa Cruz Dallas, TX, USA), permeabilized with 0.1% Triton X-100, and incubated with Odyssey Blocking buffer (LI-COR Biosciences, Lincoln, NE, USA) for 1 h. Anti-HA antibody (Santa Cruz Biotechnology, Santa Cruz, CA, USA) diluted in Odyssey Blocking Buffer was used to stain the cell monolayer at 4 °C overnight. Then, cells were washed and stained with fluorochrome-conjugated secondary antibodies together with Cell Tag (LI-COR Biosciences, Lincoln, NE, USA) (LI-COR Biosciences, Lincoln, NE, USA), a control for cell monolayer integrity, for 1 h at room temperature. After three washes with PBS (Sigma Aldrich, St. Louis, MO, USA), the plates were analyzed by the Odyssey Imaging System (LI-COR, Lincoln, NE, USA) [46]. LI-COR Image Studio software was used to determine the integrated intensities of fluorescence, and the Relative Fluorescence Unit (RFU) was expressed as a percentage compared with untreated infected cells (100%).

#### 4.2.6. Western Blot Analysis

Upon 24 h of infection and treatment, A549 cells were collected and lysed in cold RIPA buffer [20 mM Tris–HCl pH 8, 150 mM NaCl, 1% Triton X-100, 0.5% SDS and 1% sodium deoxycholate] supplemented with phenylmethyl-sulphonyl fluoride, protease inhibitor mixture, and phosphatase inhibitor (Sigma Aldrich, St. Louis, MO, USA). After 30 min of incubation, the cell lysates were centrifuged (14,000× g, 30 min, 4 °C). The supernatants obtained from the centrifugation were collected, and the protein concentration was determined by a Bradford protein assay. For the SDS-PAGE electrophoresis procedure, cell lysates samples were prepared by adding sodium dodecyl sulfate (SDS) buffer and DL-dithiothreitol. After the protein transfer, the nitrocellulose membrane was blocked with 10% milk solution (Bio-Rad Laboratories, Berkeley, CA, USA) diluted in T-TBS (0.01% Tween 20 plus Tris-buffered saline) for 1 h at room temperature and then incubated with a polyclonal goat anti-influenza antibody (anti-Flu, AB1074 Merck Millipore, Darmstadt, Germany), anti-Nrf2 (#12721 Cell Signaling) or with anti G6PD (#12263 Cell Signaling) overnight at 4 °C. After three washes with T-TBS, the membranes were incubated with a horseradish peroxidase-conjugated secondary antibody and developed using Clarity Western ECL substrate (Bio-Rad, Hercules, CA, USA). Actin was used as a loading control.

#### 4.2.7. Glutathione Assay

The total glutathione was analyzed by the Glutathione Assay Kit (ADI-900-160 Enzo) following the manufacturer’s instructions. For GSSG quantification, the samples were deproteinized with metaphosphoric acid, and an aliquot was incubated with 2-vinylpyridine to derivatize the reduced form, GSH [46]. The GSH levels were obtained by the subtraction of GSSG from the total glutathione and then normalized to the protein content of each sample determined by the Bradford method (Bio-Rad, Hercules, CA, USA).

#### 4.2.8. Immunofluorescence Analysis

A549 cells infected with PR8 virus and treated with RSV derivatives or with DMSO solvent were fixed with paraformaldehyde 4%, permeabilized with 0.1% Triton X-100, and stained with anti-NP antibody for 1 h at room temperature (Bio-Rad, Hercules, CA, USA). AlexaFlour 488-conjugated anti-mouse was used as the secondary antibody, and 4′,6-diamidino-2-phenylindole (DAPI) was used for nuclei staining [12].

#### 4.2.9. Statistical Analysis

The two-tailed Student’s test was used for statistical analyses, and a *p*-value < 0.05 was considered statistically significant. The represented data are the means of replicating experiments and the relative standard deviation (SD). GraphPad Prism™ 6.0 software (GraphPad Software Inc., San Diego, California, USA) was used for statistical analysis.

## 5. Conclusions

In conclusion, we demonstrated that nitrostilbenes, derivatives of RSV, are promising compounds with anti-influenza virus properties.

The structural manipulation of RSV, adding different substituents on the two aromatic rings and keeping the 4′-phenol moiety of RSV, changed the physicochemical properties and improved the biological activity of these new derivatives. Overall, compound **6** provided the best activity in all assays. For this reason, it represents the most promising anti-influenza agent because of its capability to drastically reduce the viral titer. We can assume that 3′-Cl has a positive effect, similar to what was previously reported [38]. However, although the compound showed a mechanism of action similar to RSV, further studies are needed to clarify whether its antiviral effect is due to an impairment of any specific cellular kinases responsible for NP phosphorylation [47,48,49].

Overall, this work demonstrates that the optimization of RSV structure can represent a good strategy to overcome the limitation of the low bioavailability of RSV. The low molecular weight, easy synthesis at a low cost, environmental impact, and high efficacy in reducing influenza virus replication at non-toxic concentrations make these compounds a good starting point in the search for new effective agents in antiviral therapy.

## Figures and Tables

**Table 1:** IC50 and CC50 values and selectivity index (SI) obtained after treatment of A549 cell line with RSV derivatives **1**–**11**. IC50: the concentration of compound causing 50% reduction in viral infection. CC50: the concentration of compound required to reduce cell viability by 50%. IC50 and CC50 are expressed in μg/mL or μmol/mL. The Selectivity Index (SI) of each compound was calculated as the ratio of CC50/IC50.

A549					
Compound	IC50		CC50		SI
	(μg/mL)	(μmol/mL)	(μg/mL)	(μmol/mL)	
**1**	20.7	0.089	87.3	0.378	4.2
**2**	13.5	0.045	39.5	0.131	2.9
**3**	12.2	0.046	30.3	0.114	2.4
**4**	14.6	0.048	38.8	0.129	2.6
**5**	23.4	0.088	59	0.223	2.5
**6**	17.1	0.062	212.3	0.770	12.4
**7**	19.5	0.080	116.1	0.481	5.9
**8**	45.1	0.203	92	0.415	2
**9**	31.7	0.104	105.5	0.466	3.3
**10**	68.3	0.277	74.1	0.300	1
**11**	1239	6.282	345	1.749	2.7

## Data Availability

Data is contained within the article and supplementary materials.

## Supplementary Materials

The following supporting information can be downloaded at: https://www.mdpi.com/article/10.3390/ph15091061/s1, Figure S1: 1H-NMR spectra (CDCl3) for (E)-2-bromo-4-(4-nitrostyryl)phenol, **12**; Figure S2: 13C-NMR spectra (CDCl3) for (E)-2-bromo-4-(4-nitrostyryl)phenol, **12**; Figure S3: 1H-NMR spectra (CDCl3) for (E)-4-(4-chlorostyryl)-2-nitrophenol, **13**; Figure S4: 13C-NMR spectra (CDCl3) for (E)-4-(4-chlorostyryl)-2-nitrophenol, **13**.

## Abbreviations

Resveratrol, RSV; glutathione, GSH; nuclear factor erythroid-2-related factor 2, Nrf2; glucose-6-phosphate dehydrogenase, G6PD; nucleoprotein, NP.
