# Supplementary material for: Nitrostilbenes: Synthesis and Biological Evaluation as Potential Anti-Influenza Virus Agents
Source: Pharmaceuticals (Basel). 2022 Aug 26;15(9):1061. doi: 10.3390/ph15091061 (PMC9505218; doi:10.3390/ph15091061)
Supplement: Supplementary file 1 [file pharmaceuticals-15-01061-s001.zip › pharmaceuticals-1832459-supplementary.pdf]

Nitrostilbenes: Synthesis and Biological Evaluation as Potential Anti-Influenza Virus Agents

Marta De Angelis [1,†], Barbara De Filippis [2,*,†], Marwa Balaha [3], Letizia Giampietro [2], Mariya Timotey Miteva [1], Giovanna De Chiara [4], Anna Teresa Palamara [1,5], Lucia Nencioni [1,*,‡] and Adriano Mollica [2,‡]

[1] Department of Public Health and Infectious Diseases, Laboratory Affiliated to Institute Pasteur Italia-Cenci Bolognetti Foundation, Sapienza University of Rome, 00185, Rome, Italy
[2] Department of Pharmacy, University "G. d'Annunzio" University of Chieti-Pescara, via dei Vestini 31, 66100 Chieti, Italy
[3] Department of Pharmaceutical Chemistry, Faculty of Pharmacy, Kafrelsheikh University, Kafr El Sheikh 33516, Egypt
[4] Institute of Translational Pharmacology, National Research Council, 00133, Rome, Italy
[5] Department of Infectious Diseases, Italian National Institute of Health, 00161 Rome, Italy
† These authors contributed equally to this work.
‡ Both co-authors acted as senior investigators and should be considered equal "last authors".
* Correspondence: barbara.defilippis@unich.it (B.D.F.); lucia.nencioni@uniroma1.it (L.N.); Tel.: +39-0871-3479-433-535 (B.D.F.); +39-0649-914-608 (L.N.)

Figure S1: ^{1}H-NMR spectra ($CDCl_3$) for (*E*)-2-bromo-4-(4-nitrostyryl)phenol, **12**

Figure S2: ^{13}C-NMR spectra ($CDCl_3$) for (*E*)-2-bromo-4-(4-nitrostyryl)phenol, **12**

Figure S3: ^{1}H-NMR spectra ($CDCl_3$) for (*E*)-4-(4-chlorostyryl)-2-nitrophenol, **13**

Figure S4: ^{13}C-NMR spectra ($CDCl_3$) for (*E*)-4-(4-chlorostyryl)-2-nitrophenol, **13**

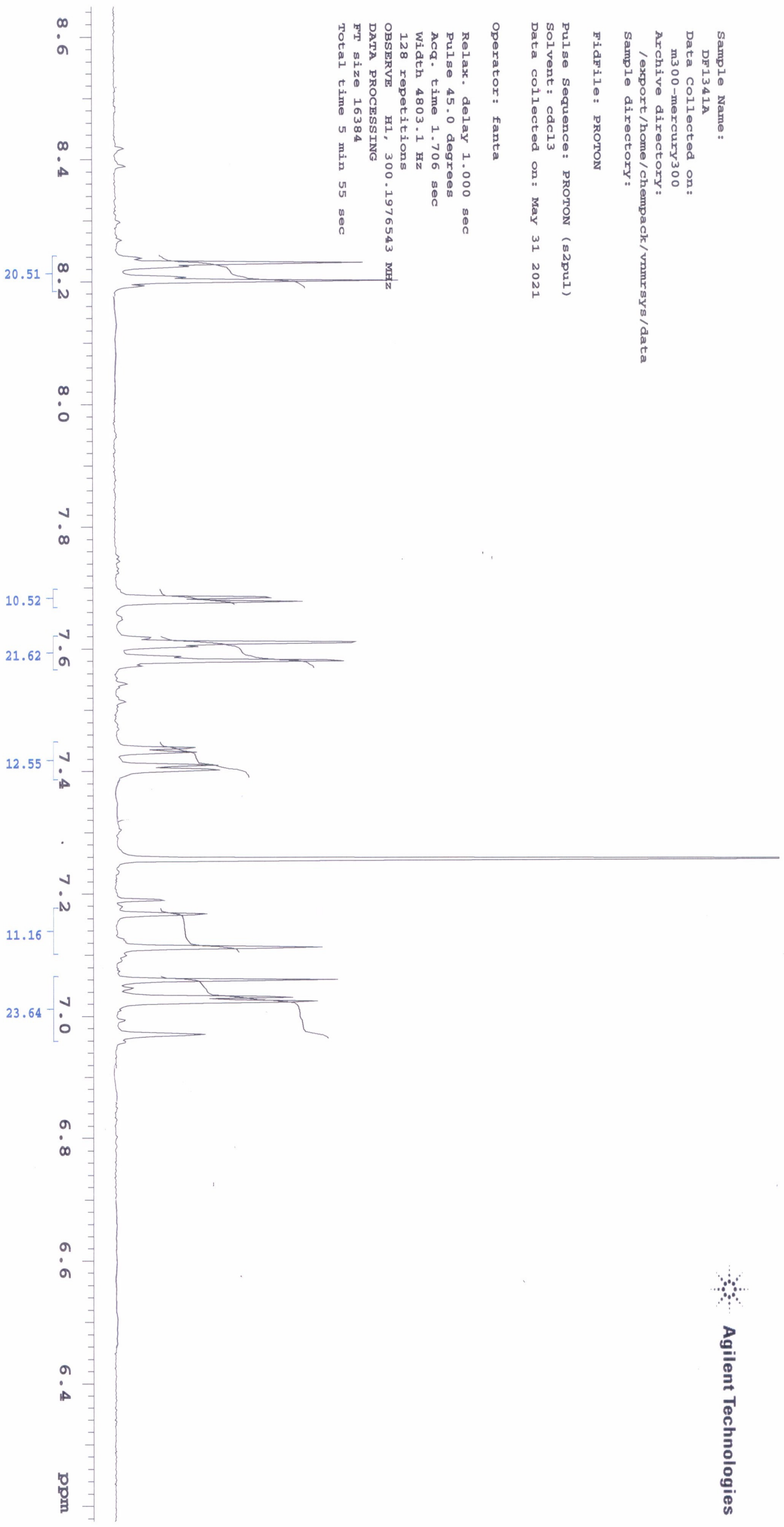

Figure S1: ^{1}H-NMR spectra ($CDCl_3$) for (*E*)-2-bromo-4-(4-nitrostyryl)phenol, **12**

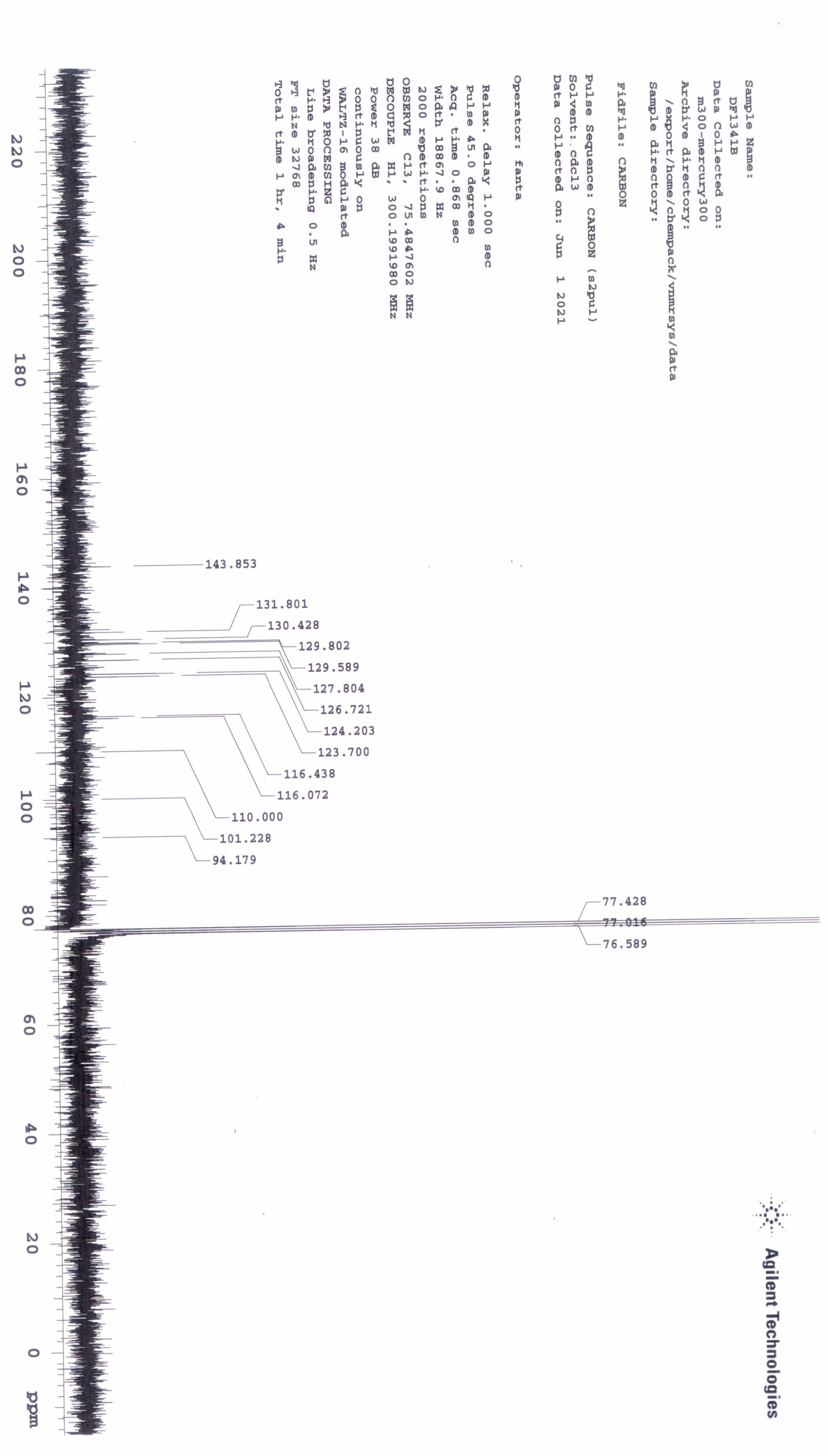

Figure S2: ^{13}C-NMR spectra ($CDCl_3$) for (*E*)-2-bromo-4-(4-nitrostyryl)phenol, **12**

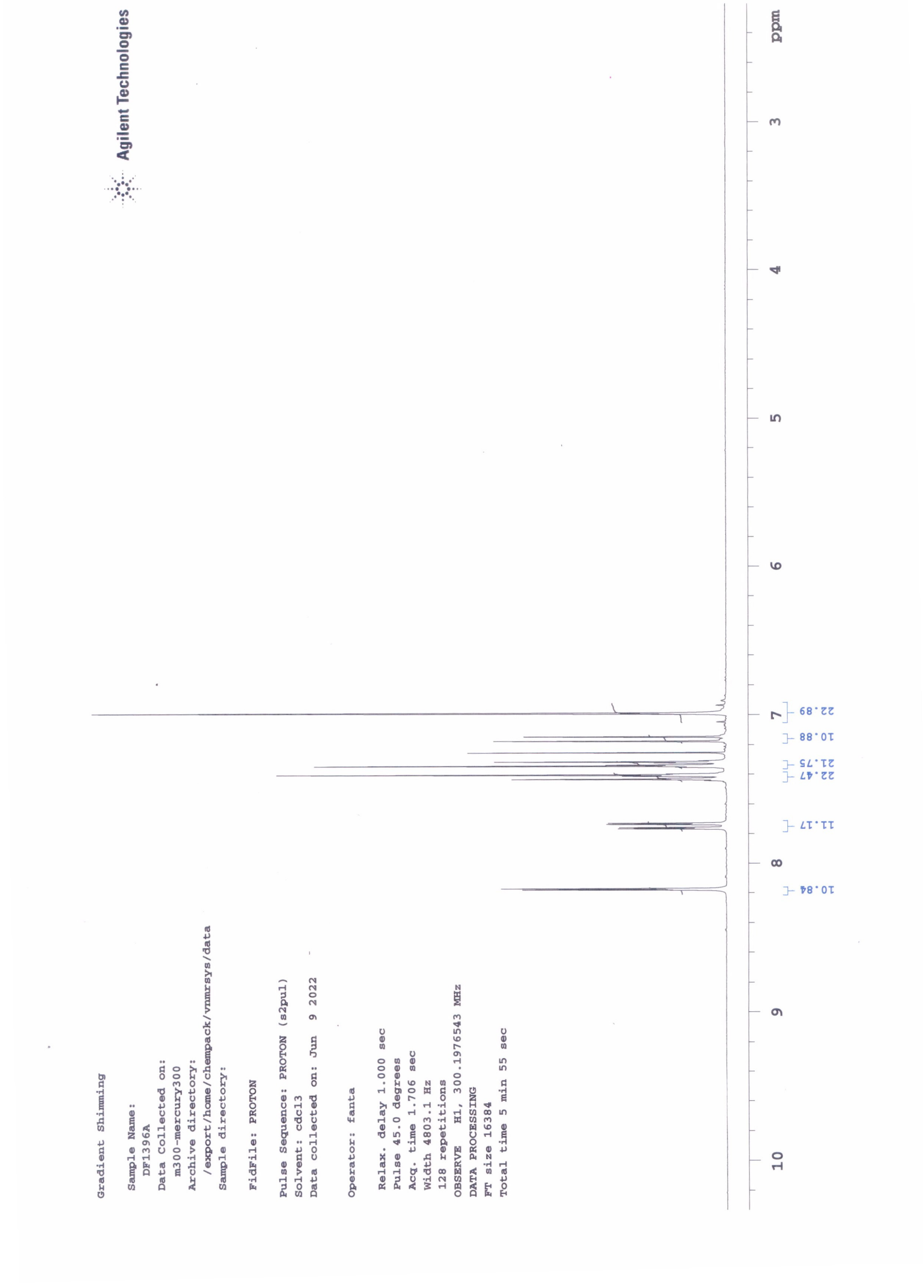

Figure S3: ^{1}H-NMR spectra ($CDCl_3$) for (*E*)-4-(4-chlorostyryl)-2-nitrophenol, **13**

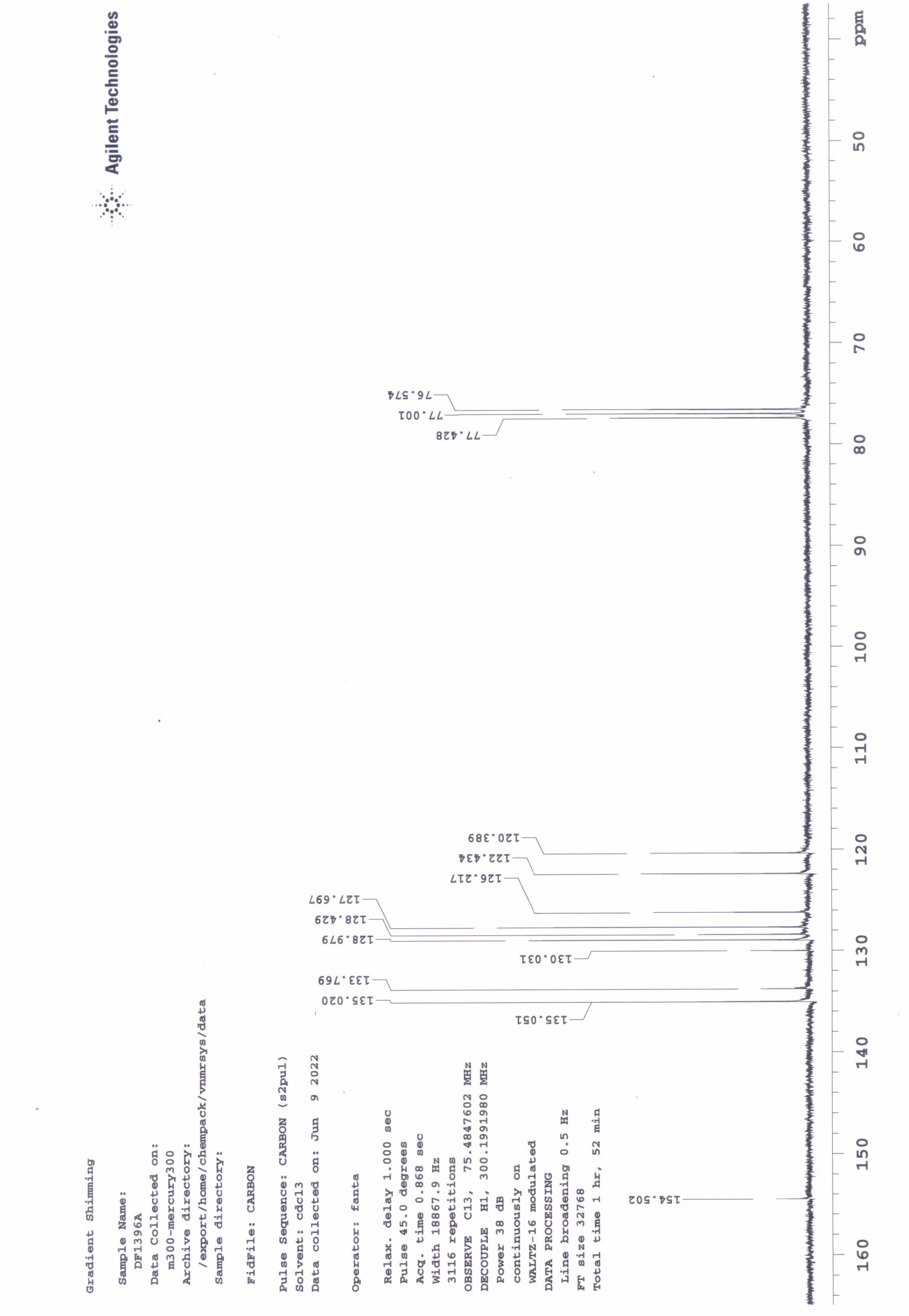

Figure S4: ^{13}C-NMR spectra ($CDCl_3$) for (*E*)-4-(4-chlorostyryl)-2-nitrophenol, **13**
